# A novel *ELOVL4* variant, L168S, causes early childhood-onset Spinocerebellar ataxia-34 and retinal dysfunction: a case report

**DOI:** 10.1186/s40478-023-01628-4

**Published:** 2023-08-11

**Authors:** Yeboah Kofi Gyening, Keren Boris, Mignot Cyril, Richard S. Brush, Marie-Cécile Nassogne, Martin-Paul Agbaga

**Affiliations:** 1 Department of Cell Biology, University of Oklahoma Health Sciences Center, DMEI 423 Parke Pavilion, 608 Stanton L. Young Boulevard, Oklahoma City, OK 73104 USA; 2 Department of Ophthalmology and Dean McGee Eye Institute, University of Oklahoma Health Sciences Center, Oklahoma City, USA; 3 UF de Génomique du Développement, Centre de Génétique Moléculaire et Chromosomique, AP-HP.Sorbonne Université - Hôpital de La Pitié-Salpêtrière, Paris, France; 4 Unité Déficiences Intellectuelles/Troubles du, Développement, Service de Génétique Clinique et Médicale, AP-HP. Sorbonne Université -Hôpital de La Pitié-Salpêtrière, Paris, France; 5 Centre de Référence des Maladies Héréditaires du Métabolisme - Service de NeurologiePédiatrique, Cliniques Universitaires Saint-Luc –UCLouvain, Avenue Hippocrate, 10, 1200 Brussels, Belgium

**Keywords:** Spinocerebellar ataxia 34 (SCA34), Elongation of very long chain fatty acids-4 (ELOVL4), Very long chain polyunsaturated fatty acid (VLC-PUFA), Very long chain saturated fatty acid (VLC-SFA), Erythrokeratodermia variabilis (EKV), Autosomal dominant Stargardt-like macular dystrophy (STGD3)

## Abstract

Spinocerebellar ataxia 34 (SCA34) is an autosomal dominant inherited disease characterized by age-related cerebellar degeneration and ataxia caused by mutations in the *Elongation of Very Long Chain Fatty Acid-4* (*ELOVL4)* gene. The ELOVL4 enzyme catalyzes the biosynthesis of both very long chain saturated fatty acids (VLC-SFA) and very long chain polyunsaturated fatty acids (VLC-PUFA) that are important for neuronal, reproductive, and skin function. Several variants in *ELOVL4* have been shown to cause different tissue-specific disorders including SCA34 with or without Erythrokeratodermia Variabilis (EKV), a skin condition characterized by dry, scaly skin, Autosomal Dominant Stargardt-Like Macular Dystrophy (STGD3), and seizures associated with neuro-ichthyotic disorders. What is puzzling is how different mutations in the same gene seem to cause different tissue-specific disorders. To date, no SCA34 patients have presented with both SCA34 and STGD3 pathology that is caused by *ELOVL4* variants that cause truncation of ELOVL4. Here, we report a novel case of an early childhood onset and rapidly progressive cerebellar degeneration and retinal dysfunction in a Belgian-Italian girl who developed severe dysarthria and gait problems starting at about 3.5 years of age and progressed to immobility by 4.5 years of age. Brain magnetic resonance imaging (MRI) revealed progressive vermian, cerebellar, cortical atrophy, progressive corpus callosum slimming, and hot cross bun sign visible on the MRI. Ophthalmological examinations also revealed progressive macular dysfunction as measured by electroretinography. Using exome sequencing, we identified a novel heterozygous *ELOVL4* variant, c.503 T > C (p. L168S) in the patient. To understand the enzymatic function of this novel ELOVL4 variant and how it alters the levels of VLC-PUFA and VLC-SFA biosynthesis to contribute to cerebellar and retinal dysfunction, we expressed wild-type *ELOVL4* or the L168S *ELOVL4* variant in cell culture and supplemented the cultures with VLC-PUFA or VLC-SFA precursors. We showed that the L168S ELOVL4 variant is deficient in the biosynthesis of VLC-SFA and VLC-PUFA. Our work suggests that differential depletion of these fatty acids may be a contributing factor to the pathogenic mechanism of SCA34 with or without EKV. Further studies will help further define how the different ELOVL4 variants cause different tissue-specific disorders with variable ages of onset.

**Supplementary Information:**

The online version contains supplementary material available at 10.1186/s40478-023-01628-4.

## Introduction

Spinocerebellar Ataxias (SCAs) are a genetically diverse group of autosomal dominant inherited disorders marked by age-dependent loss of balance, loss of gait, and slurred speech [1]. Currently, there are more than 40 genes known to cause different types of SCAs that are usually characterized by damage to different brain regions, especially to Purkinje neurons in the cerebellum followed by cerebellar atrophy [2–5]. In addition, cognition and mobility of individuals with SCA are impaired, and many SCAs lead to premature death [6, 7]. In the last decade, several different heterozygous point mutations in *the Elongation of Very Long Chain Fatty Acids-4 (ELOVL4)* have been reported to cause Spinocerebellar Ataxia-34 (SCA34) [8–14]. An *ELOVL4* variant [c.540G > C (p. L168F)] reported in a large French-Canadian family in 2014 was the first heterozygous *ELOVL4* variant shown to cause SCA34 pathology with or without Erythrokeratodermia Variabilis (EKV) [13]. Magnetic resonance imaging (MRI) of the brain of affected patients revealed severe atrophy of the cerebellum and the pons [13]. Following this initial report, other SCA34-causing *ELOVL4* variants, c.736 T > G (p. W246G), c.539A > C (p. G180P), c.512 T > C (p. I171T), and c.698C > T (p. T233M), have been reported to cause the disease with or without EKV [9–12]. The EKV pathologies were reported in patients carrying the c.540G > C (p. L168F), c.539A > C, p. G180P, and c. 698C > T, p T233M. In 2020, further characterization of the C.504G > C p. L168F variant in another French-Canadian family revealed that none of the nine clinically affected family members had EKV [8]. This may indicate incomplete penetrance of EKV in contrast to the SCA34 pathology or environmental and nutritional factors contributing to EKV pathology. To date, no patients with *ELOVL4* variants that developed SCA34 with or without EKV were reported to have macular degeneration as reported in STGD3 patients. However, it must be noted that an *ELOVL4* variant, c.512 T > C, p. I171T, was recently shown to cause SCA34 with retinal abnormalities consistent with retinitis pigmentosa in four of eight individuals that carry the mutation [9]. Except for c.736 T > G (p. W246G) and c. 698C > T, p T233M variant, which occur in exon 6 like other ELOVL4 variants that cause STGD3, all the other heterozygous SCA34-causing mutations occur in exon 4 of *ELOVL4* [14]. The critical role of ELOVL4 function in health and disease is further supported by the fact that inheritance of homozygous *ELOVL4* variants causes severe pathologies that affect the brain and skin resulting in early childhood mortality [15, 16]. Inheritance of the homozygous *ELOVL4* variant, c.78C > G; p. Tyr26 ∗ and c.646C > T, p. Arg216X (41) located in exon 5, and c.690del p. Ile230Metfs ∗ 22 in exon 6, causes intellectual disability, seizures, hypertonia, and premature death [15, 16], which underscores the critical role of functional ELOVL4 for normal growth and survival.

ELOVL4 is an essential enzyme that catalyzes the first and rate limiting step in the elongation of both saturated and polyunsaturated fatty acids to produce VLC-SFAs and VLC-PUFAs containing 28 or more carbons that we collectively call very long chain fatty acids (VLC-FA) [17]. In the retina, depletion/loss of VLC-PUFAs enriched in retinal phosphatidylcholines (PCs) leads to a significant decline in visual function [18, 19]. On the other hand, VLC-SFAs incorporated into sphingolipids are the predominant ELOVL4 product in the brain and are necessary for proper brain health [20]. We showed that VLC-SFA are enriched in synaptic vesicles where they regulate presynaptic transmitter release kinetics in cultured mouse hippocampal neurons [20] and that the W246G variant that suppresses VLC-SFA biosynthesis both in vitro [21] and in the skin [22] also impairs synaptic plasticity in parallel and climbing fibers in the cerebellum to cause motor defects in a rat model of SCA34 [23].

For most SCA34 patients, the onset of ataxia occurs in the third or fourth decade of life, although reports of childhood-onset EKV and ataxia have been reported in some other SCAs [14, 24]. Here, we report a case of childhood-onset SCA34 with retinal dysfunction resulting from a novel *ELOVL4* variant (NM_022726.3: c.503 T > C, p. L168S). The patient developed progressive cerebellar and cortical atrophy and gait starting at 3.5 years of age. A year later, the patient lost her ability to walk. In addition to brain pathology, she also developed progressive retinal dysfunction, especially at the macular level as measured by electroretinography (ERG), at 4.5 and 6.5 years of age. Using in vitro characterization assays, we showed that the L168S ELOVL4 variant is enzymatically deficient in the biosynthesis of both VLC-SFA and VLC-PUFA, which are necessary for normal brain and retinal function [20, 25]. These findings suggest that the reduction in both VLC-SFA and VLC-PUFA biosynthesis may be a contributing factor to the pathogenic mechanism of SCA34 and retinal dysfunction in this patient [21].

## Case presentation

A four-year-old girl was brought to the clinic because of problems with gait imbalance and deterioration in her ability to walk. Her family history was unremarkable. She was the first child of unrelated Belgian-Italian parents, both of whom are not affected. The pregnancy, delivery, and neonatal period were uneventful. She said her first words at 12 months and made little progress thereafter. Because she was not able to form complete sentences, she was referred to a speech therapist for treatment where she was diagnosed with dysarthria. She started walking at 17 months of age, but her parents noticed gait problems and deterioration in her walking starting at about 3.5 years of age. She had problems running and climbing stairs. By the age of 4.5 years, she was unable to walk by herself. However, her weight, height, and head circumference were all within the normal range. There were no dysmorphic signs. The child had axial hypotonia, an ataxic gait, Achilles tendon retractions, positive Gowers' sign, brisk deep-tendon reflexes, and Babinski sign, which suggests upper motor neuron lesion mostly due to defects in the pyramidal system [26, 27].

**Fig. 1:**
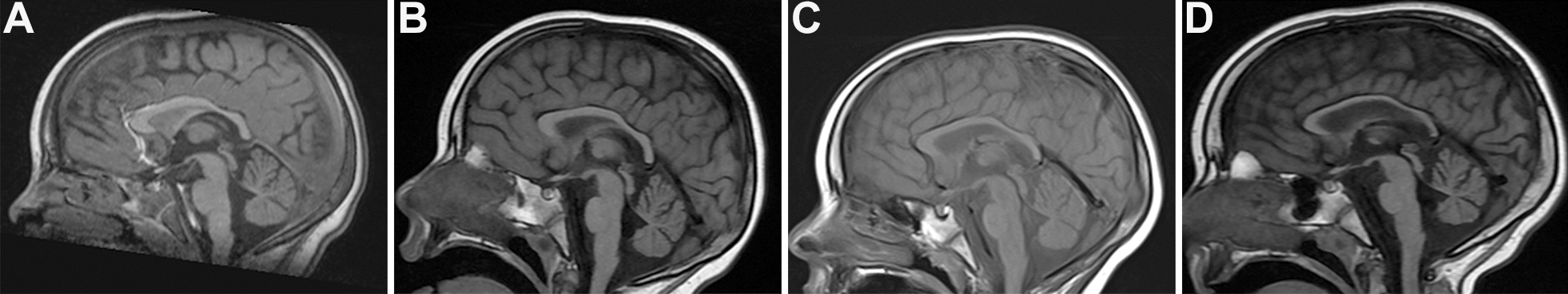
Brain magnetic resonance imaging (MRI) performed at **A** 4 years, **B** 4 years and 5 months, **C** 5 years and 2 months, and **D** 7 years and 6 months of age

**Fig. 2:**
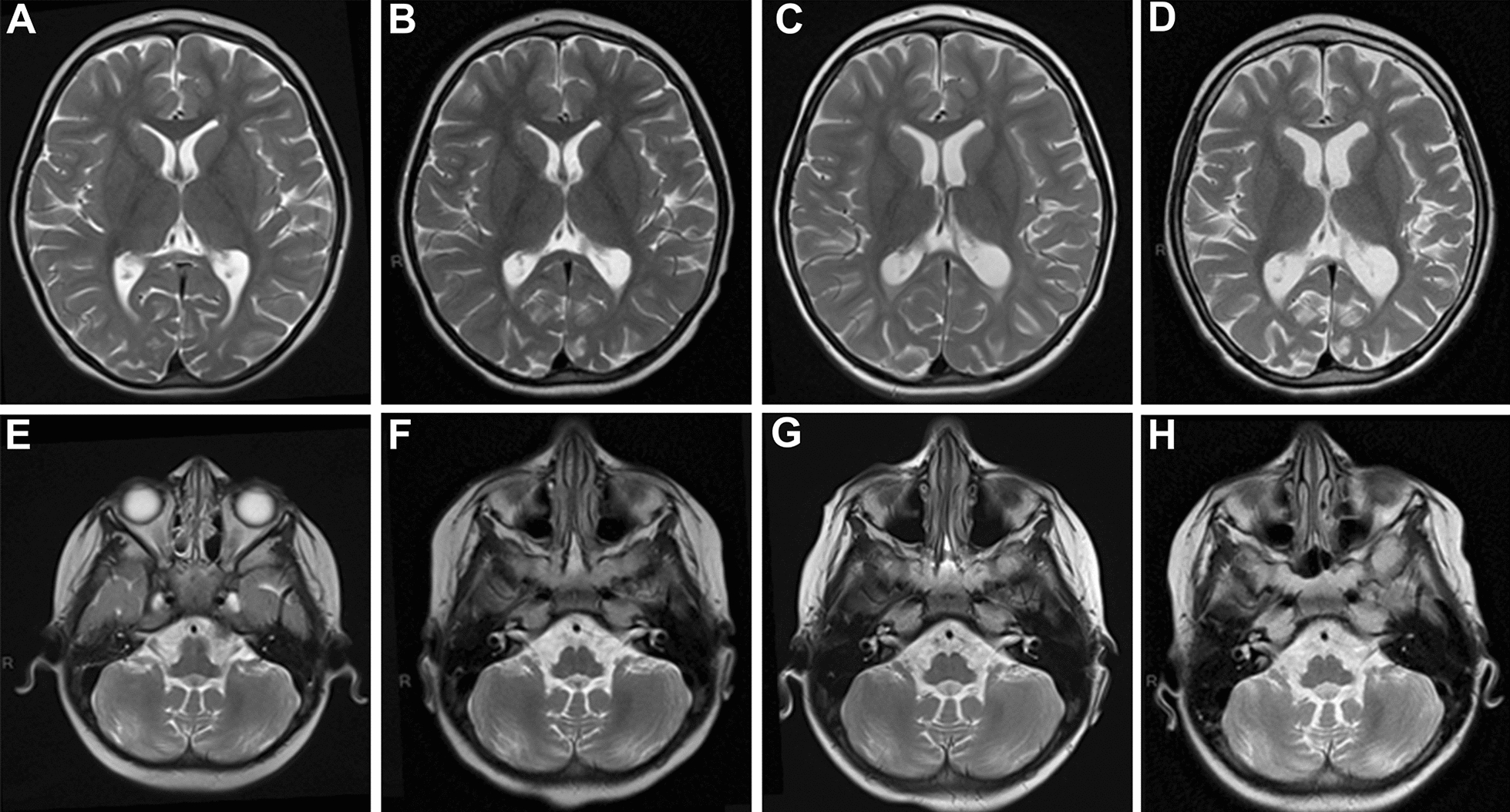
Brain magnetic resonance imaging (MRI) performed at **A** and **E** 4 years, **B** and **F** 4 years and 5 months, **C** and **G** 5 years and 2 months, and **D** and **H** 7 years and 6 months of age. T2 axial and T1 sagittal sequences reveal progressive cerebellar and cortical atrophy with slimming of corpus callosum

Brain magnetic resonance imaging (MRI) revealed mild vermian atrophy at the age of 4 years (Figs. 1 and 2). Subsequent brain MRI confirmed progressive cerebellar and cortical atrophy and progressive corpus callosum slimming with visible hot cross bun sign (Figs. 1 and 2). Cerebrospinal fluid (CSF) cytology, protein, glucose, and lactate levels were normal. Additional metabolic investigations, including peroxisomal, lysosomal, and purine analyses, yielded normal results. Electroencephalography (EEG), nerve conduction velocities, brainstem auditory-evoked responses, and kidney and heart ultrasound were normal.

Her initial ophthalmological examination was normal, except for a vertical and horizontal optokinetic nystagmus. However, electroretinography examinations performed at the age of 4.5 years showed attenuation of rod and cone conduction predominantly at the macular level. Her clinical evolution consisted of progressive motor decline with severe axial hypotonia with absence of sitting position, few spontaneous movements, dysmetria, and severe dysarthria. Further ERG analyses at the age of 6.5 years confirmed retinal dysfunction as specified by the International Society for Clinical Electrophysiology of Vision (ISCEV) standard for full field clinical ERG [28, 29] (Additional file 1: Fig. 1A–F and Additional file 2: Fig. 2A–F). At 4.5 years of age, the patient had below-normal and delayed responses to the dark-adapted (DA) 0.01 and 3.0 ERGs (Additional file 1: Fig. 1A, B). The light-adapted (LA) 3.0 response was delayed and showed below normal amplitude whereas the flicker response had a normal amplitude that was delayed (Additional file 1: Fig. 1D, F). These results indicate a pan retinal defect in both the rod and cone systems with the rods having slightly greater dysfunction than the cones. Two years later at age 6.5, the DA at 0.01 was undetectable and the DA 3.0 ERG was delayed and severely reduced below normal (Additional file 2: Fig. 2A–D). There was no change in the LA responses (LA 3.0 was still reduced and delayed; while the flicker amplitude was normal but delayed) (Additional file 2: Fig. 2E, F). Altogether the ERG results showed that the rods were likely the primary photoreceptor type that were affected by disease with profound loss of the DA responses within the two years’ time. Unfortunately, the child died at the age of 10 after the placement of a gastrostomy. However, autopsy was not done on the patient after her death to enable us have further pathological insight into how the L168S ELOVL4 variant contributed to her neurological symptoms.

**Fig. 3:**
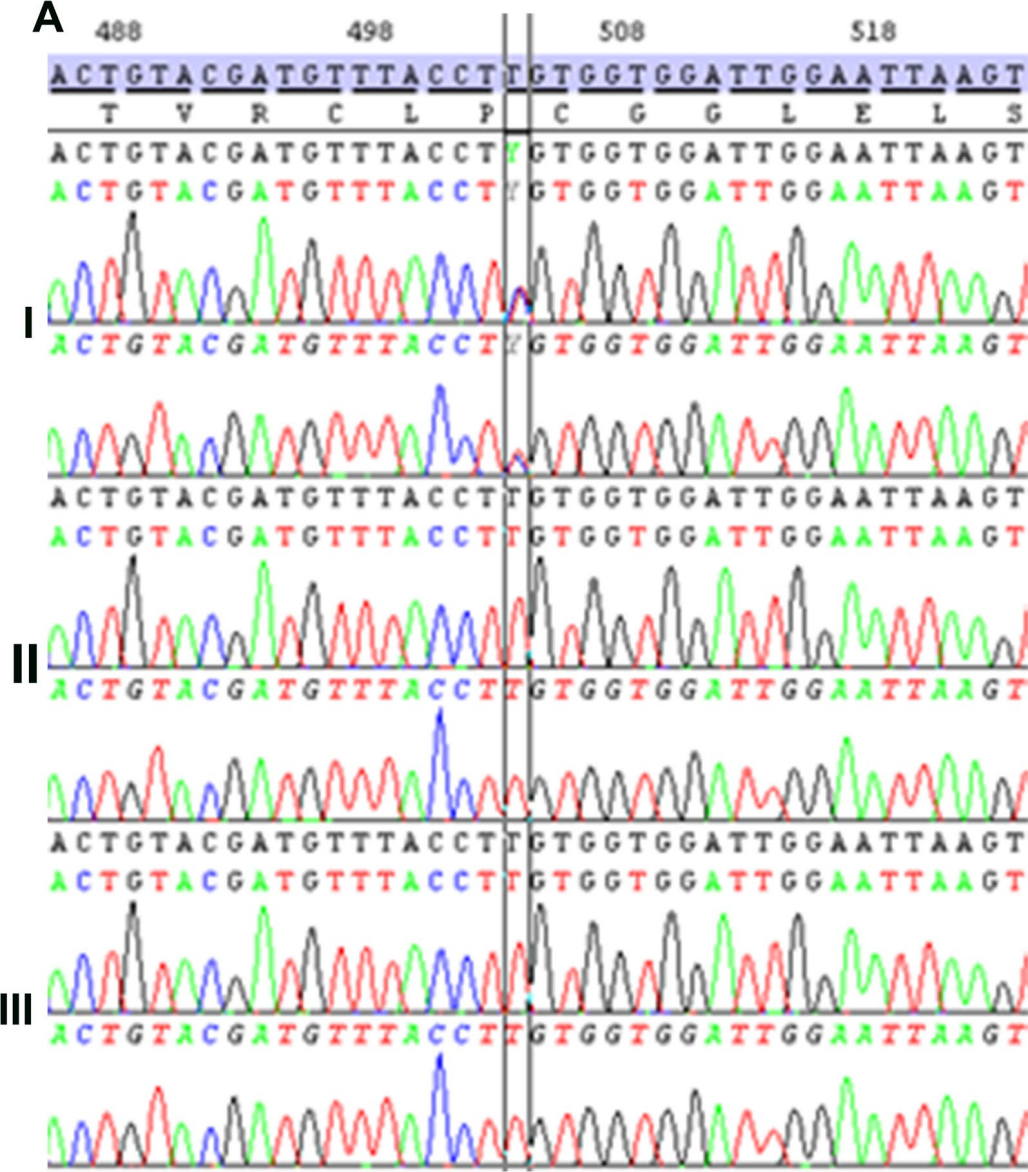
Confirmation of the NM_022726.3: c.503 T > C de novo variant with Sanger sequencing. (I) index case, (II) father, (III) mother with forward primers (upper electropherogram) and reverse primers (lower electropherogram). The variant is between black lines on the 503 position

To determine what contributes to the observed phenotypic and pathological signs in the patient, exome sequencing was performed, which identified and confirmed a de novo ELOVL4 variant, c.503 T > C (p. Leu168Ser), in the coding region of *ELOVL4* (MIM *133190) (Fig. 3). Since this novel *ELOVL4* variant is similar to the L168F variant, except for replacement of phenylalanine with serine at the conserved lysine 168 position in ELOVL4, we carried out further in vitro biochemical analyses to determine how this novel ELOVL4 variant contributes to the early-onset progressive brain and retina disorders of the patient.

## In vitro biochemical characterization: L168S SCA34 variant suppresses VLC-SFA and VLC-PUFA biosynthesis

**Fig. 4:**
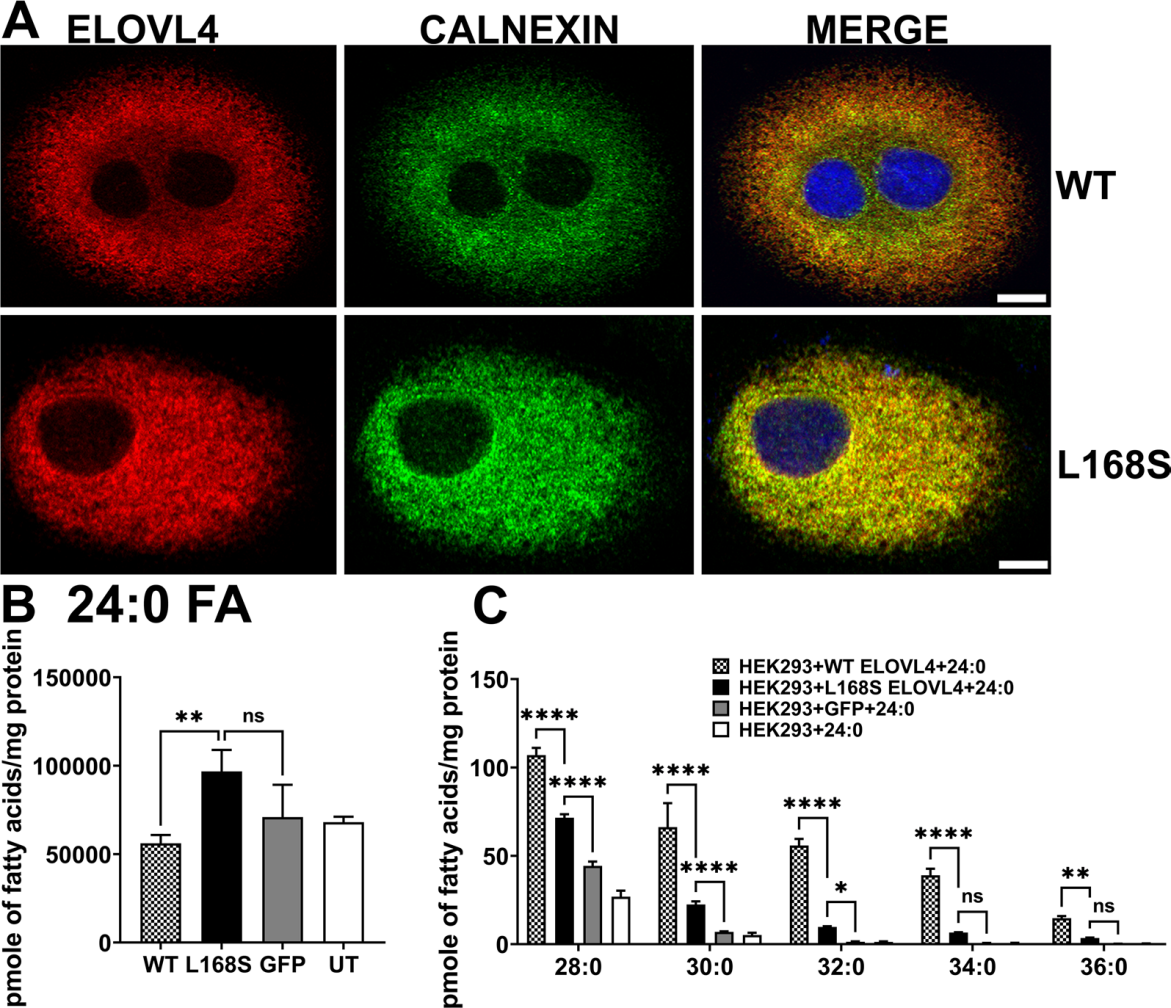
L168S ELOVL4 localizes properly to the Endoplasmic Reticulum (ER) but is deficient in Very Long Chain Saturated fatty acid synthesis. **A** ARPE-19 cells transduced with WT mouse *ELOVL4* and L168S mouse *ELOVL4 MYC* constructs were immunostained for MYC (red) and CALNEXIN (green). WT and L168S colocalizes with ER transmembrane marker Calnexin (Scale bar, 10 µm) indicating their localization to the ER necessary for their biochemical activity. **B** Accumulation of 24:0 (lignoceric fatty acid) in HEK293 cells expressing L168S mutant protein. HEK293 cells overexpressing WT, L168S and control cells (GFP and UT) were supplemented with 24:0 SFA for 72 h. **C** L168S showed enzymatic activity by making higher amounts of 28:0, 30:0 and 32:0 compared to GFP controls. However, L168S synthesized lower levels of VLC-SFA levels compared to WT. Results are the mean ± SD (n = 3). Statistical significance was assessed for **B** and **C** by ANOVA with Tukey’s post-hoc test. *p < 0.05; **p < 0.01; ***p < 0.001; ****p < 0.0001; ns not significant in comparison with WT

The L168S variant, similar to the L168F ELOVL4 variant, is six amino acid residues away from the histidine catalytic core (HXXHH) that is essential for the biosynthesis of both VLC-SFA and VLC-PUFA by ELOVL4 [14, 30, 31]. Beaudin et al., reported mislocalization of ELOVL4 L168F variant beyond the perinuclear region with a punctate and aggregated appearance in fibroblast cells from L168F patients [8]. To explore the impact of the L168S variant on ELOVL4 subcellular localization as well as VLC-PUFA and VLC-SFA biosynthesis, we generated adenoviral particles of Myc-tagged wild-type (WT) mouse *Elovl4* and Myc-tagged L168S mouse *Elovl4*. Immunofluorescence studies after transducing ARPE-19 cells with the mouse WT *Elovl4* and the L168S *Elovl4* showed that WT and L168S proteins co-localized with the endoplasmic reticulum (ER) marker Calnexin, indicating their localization in the ER (Fig. 4A). This localization pattern is different from what was reported by Beaudin et al. [8] for the L168F variant but is consistent with our recent results for the L168F localization in vitro [21]. Our result is further consistent with the fact that the L168S ELOVL4 is a full-length protein with intact ER retention/retrieval signal, so we expected it to be localized to the ER membrane.

Since the L168S variant seems to impair neuronal health and VLC-SFAs enriched in neuronal tissues are necessary for normal neuronal function [20, 32], and the L168S ELOVL4 variant was targeted to the ER, the site of VLC-FA biosynthesis, we analyzed the effect of the L168S variant on VLC-SFA synthesis. We overexpressed WT and L168S as well as GFP in HEK293 cells and supplemented the cells with the VLC-SFA precursor, 24:0 for 72 h followed by lipid extraction and analysis using gas chromatography-mass spectrometry. Our results showed a significant uptake of 24:0 FA in L168S overexpressing cells (Fig. 4b). Despite uptake of the 24:0, the L168S ELOVL4 variant consistently synthesized significantly lower amounts of VLC-SFA products 28:0, 30:0, 32:0, 34:0, and 36:0, compared to WT. Compared to the GFP control cells, L168S ELOVL4 seemed to retain some level of enzymatic activity since it synthesized significant amounts of 28:0 and 30:0, the major VLC-SFA enriched in synaptic vessels [20], as well as 32:0 (Fig. 4C).

**Fig. 5:**
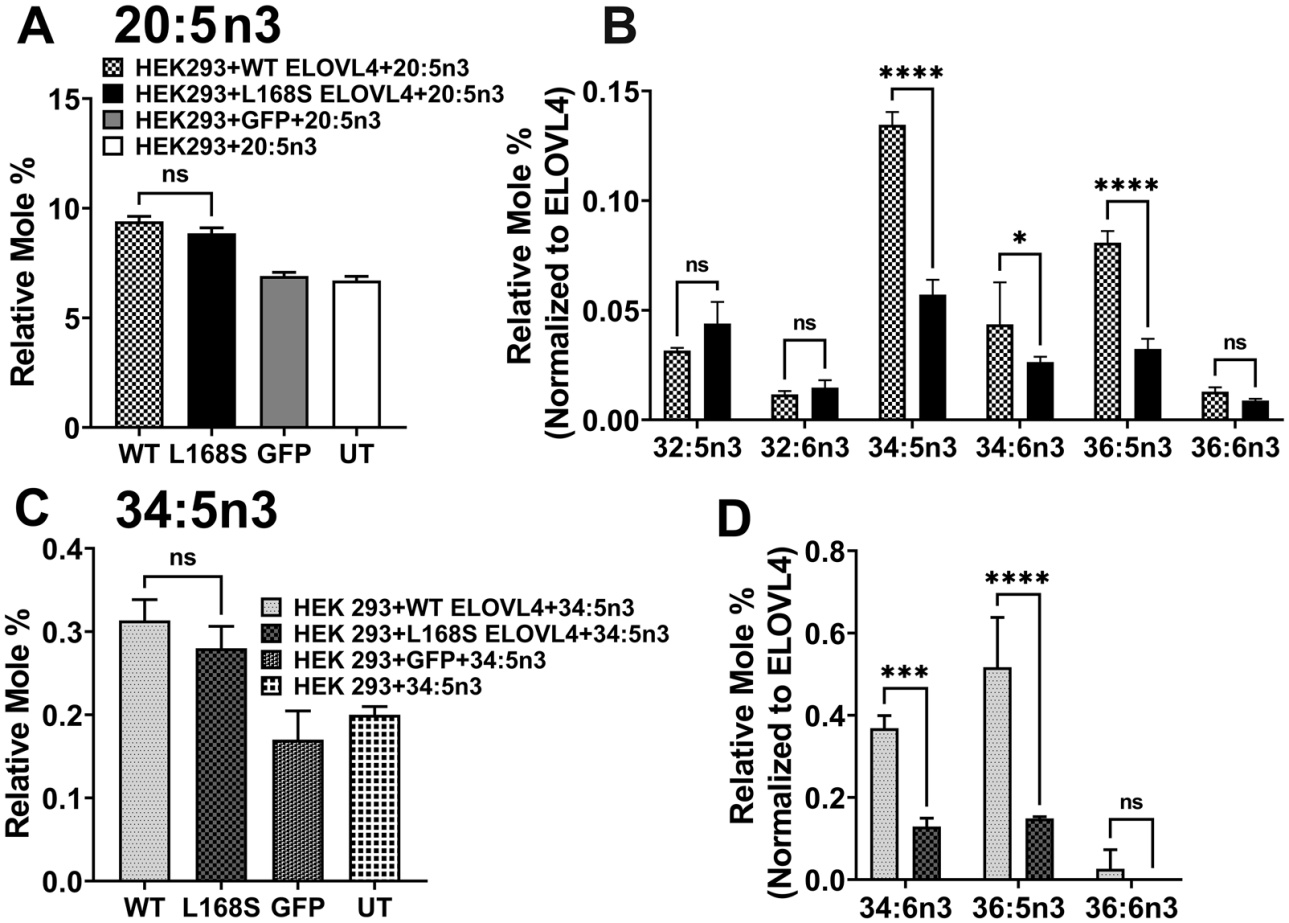
L168S ELOVL4 variant is deficient in VLC-PUFA synthesis. **A** Uptake of 20:5n3 after 72 h in HEK293T cells overexpressing WT, L168s and control cells (GFP and UT) supplemented with 20:5n3. **B** Relative mole% of VLC-PUFA levels normalized to WT ELOVL4 and L168S ELOVL4 protein levels after 20:5n3 supplementation. L168S mutation decreased the synthesis of the major VLC-PUFA 34:5n3 followed by 36:5n3 found in human retina. **C** Uptake of 34:5n3 after 72 h in HEK293T cells overexpressing WT, L168S and control cells (GFP and UT) supplemented with 34:5n3. **D** Elongated products of 34:5n3 normalized to WT ELOVL4 and L168S ELOVL4 expression levels. L168S ELOVL4 protein was deficient in catalyzing the addition of two carbons to produce 36:5n3 similar to cells treated with 20:5n3. Results are the mean ± SD (n = 3). Statistical significance was assessed for **A**, **B**, **C**, **D** by ANOVA with Tukey’s post-hoc test. *p < 0.05; **p < 0.01; ***p < 0.001; ****p < 0.0001; ns not significant in comparison with WT

VLC-PUFA are necessary for retinal function [25, 33]. Considering the progressive decline in retinal function in the patient, we determined the effect of the L168S ELOVL4 on VLC-PUFA synthesis by supplementing HEK293 cells expressing Myc-tagged WT and L168S ELOVL4 with the VLC-PUFA precursor 20:5n3 (EPA) [30, 31, 34]. Our results showed an equivalent uptake of 20:5n3 in both WT and L168S ELOVL4-expressing cells (Fig. 5A). However, compared to WT ELOVL4-expressing cells, L168S ELOVL4 was deficient in biosynthesis of 34:5n3, 34:6n3, and 36:5n3 (Fig. 5B**)**. We did not detect any VLC-PUFA products in both GFP and untransduced (UT) controls (Fig. 5B), which again supports the fact that the L168S ELOVL4 that is targeted to the ER retains some enzymatic activity in contrast to the 5-bp STGD3 mutant ELOVL4 that lacks VLC-PUFA biosynthesis due to loss of its ER localization signal [31]. To further characterize the enzymatic function of the L168S ELOVL4 towards elongation of VLC-PUFAs, we supplemented HEK293 cells expressing either WT or L168S ELOVL4 with 34:5n3, a direct substrate of ELOVL4. Uptake of 34:5n3 VLC-PUFA was equivalent in L168S and WT overexpressing cells (Fig. 5C). However, the L168S ELOVL4 was deficient in making 34:6n3 and 36:5n3 compared to WT (Fig. 5D**)**. Taken together, results from these biochemical analyses suggest that even though the L168S ELOVL4 is targeted to the ER, the substitution of serine for leucine in L168S ELOVL4 has a significant impact on biosynthesis of the full complement of both VLC-PUFA and VLC-SFA products that are necessary for retinal and brain function.

## Discussion and conclusions

Here, we report the first case of a childhood-onset SCA34 with retinal dysfunction resulting from a novel *ELOVL4* variant (NM_022726.3: c.503 T > C, p. L168S) that also leads to death within the first decade of life as previously reported [15]. We provide detailed biochemical analyses of the L168S ELOVL4 enzyme activity in the biosynthesis of VLC-SFA and VLC-PUFA products and show that the L168S ELOVL4 is deficient in biosynthesis of both VLC-SFA and VLC-PUFA. In SCA34 patients, the main pathological finding is age-related cerebellar atrophy as determined by MRI [8–14]. Indeed, MRI scans of the brain of the patient showed not only progressive cerebellar atrophy but also progressive cortical and vermian atrophy with corpus callosum thinning with hot cross bun signs at a very early age of 4 years. Consistent with other *ELOVL4* variants such as T233M and W246G carrying patients that do develop cerebellar atrophy but do not have EKV [12, 35], this patient did not develop any skin conditions or ichthyosis. However, disease progression was very rapid with the patient losing her ability to walk within a year of first developing gait problems at 3.5 years. Childhood onset SCAs have been reported in SCA2, SCA7, and SCA13 [36, 37], but this is the first report of early child-onset SCA34 caused by  an *ELOVL4* variant that also affects macular function and caused early-childhood death. Our biochemical analyses showed that L168S ELOVL4 variant localizes to the ER, which is necessary for its function [30]. Using both ELOVL4 and MYC antibodies, we did not detect any mislocalization of the L168S ELOVL4 as reported in fibroblast cells from L168F patients [8]. Despite localization to the ER, the L168S variant showed consistent deficiency in VLC-SFA synthesis as we recently reported for W246G and L168F variants of ELOVL4 proteins [21]. In addition, the L168S variant was also deficient in biosynthesis of 34:5n3 and 36:5n3, which are the predominant VLC-PUFAs found in the human retina [18]. These results further suggest that defects in neuronal VLC-FA biosynthesis may contribute to the mechanism by which the different *ELOVL4* variants contribute to SCA34 pathology and that mutations that severely affect both VLC-SFA and VLC-PUFA biosynthesis have detrimental effects on health and childhood quality of life and survival.

The L168F *ELOVL4* variant was the first *ELOVL4* variant known to cause SCA34 [13]. Interestingly, the L168S occurs at the same amino acid residue, which is six amino acids from the critical catalytic site of ELOVL4. Although the present case involves a single patient, none of the 19 affected French-Canadian families carrying the L168F variant had early childhood onset of ataxia and cerebellar degeneration [13]. The earliest onset of ataxia reported for the L168F variant was at 35 years of age with the individual developing mild symptoms associated with SCA [13]. The early childhood onset of brain and retinal pathologies in the L168S variant and the rapid progression of disease pathologies and death of this patient within the first decade of life even though not related to her symptoms suggest critical structural changes due to the amino acid substitution that may influence disease progression. Even though pathological examinations were not done on the patient after her death, based on the American College of Medical Genetics and Genomics criteria (with the help of Intervar), the variant is predicted to be pathogenic. Using bioinformatic tools, Polyphen2, the L168S variant, which is at a highly conserved amino acid residue, was predicted to be functionally deleterious and could be more damaging with a score of 1.00 (sensitivity: 0.00; specificity: 1.00) compared to the previously published L168F that was predicted to be possibly damaging with a score of 0.867 (sensitivity: 0.83; specificity: 0.93) [13] (http://genetics.bwh.harvard.edu/pph2/). In addition, mutation taster predicted both L168F and L168S variant as disease-causing with PROVEAN predictions of L168F (-2.767) and L168S (-4.083) as deleterious (https://www.mutationtaster.org/). As such, it was not surprising that the L168S variant patient had much more severe disease onset and rapid progression compared to other SCA34-causing *ELOVL4* variants. For example, a patient carrying the T233M *ELOVL4* variant was reported to develop ataxia starting at 15 years of age [10]. However, at the time of examination of this patient at 60 years of age, an MRI of the brain showed only subtle flattening of the ventral pons and mild cerebellar atrophy [10]. Another patient carrying the Q180P *ELOVL4* variant developed ataxia in his mid-20 s and showed cerebellar and pontine atrophy [11]. Japanese patients also carrying the W256G variant developed gait ataxia between 13–56 years of age [12]. However, disease progression was reported to be very slow, and patients did not require assistance with walking with a walker or cane until the age of 60 years or older [12]. Taken together, it looks like the nature of the mutation and its effect on normal ELOVL4 function most likely through defects in VLC-FA biosynthesis or conformational changes in protein structure are critical to disease onset and severity of the pathologies.

Similar to some individuals with the L168F variant [13], the L168S patient had dysarthria. What is more revealing about the L168S patient is that she also showed progressive retinal dysfunction predominantly in the macula as measured by ERG. To rule out other potential inherited retinal degeneration gene(s) that could contribute to the patient’s retinal dysfunction, we reviewed the patient’s gene panel for potential retinal degeneration mutations that could cause the retinal pathology. However, we did not find any mutations that could account for the retinal pathology except for the L168S *ELOVL4* variant. Studies have extensively shown the role of ELOVL4 and VLC-PUFA in retinal function and photoreceptor survival, although the mechanism has not been fully elucidated [14, 25, 31]. Thus, defects in VLC-PUFA biosynthesis and depletion in the retina in the patient presented here may be the reason for the retinal dysfunction since the L168S variant lacks VLC-PUFA biosynthesis. Despite the importance of ELOVL4 and VLC-PUFA in the retina, lack of information on ophthalmologic tests have made it difficult to know whether other *ELOVL4* variants that cause SCA34 also cause retinal dysfunction. For instance, no comprehensive retinal tests have been conducted or reported for patients with the L168F ELOVL4 variant [13]. Except for the c.512.T > C, pIle171Thr variant that causes SCA34 and retinitis pigmentosa [9], all the other SCA34 patients reported so far in the literature did not undergo any retinal electrophysiological tests [8, 10–12]. Therefore, SCA34 patients carrying the various *ELOVL4* variants may have impaired retinal function even in the absence of retinal degeneration at the time of their clinical examination. The possibility of late-onset clinical retinal changes could occur later in life in some SCA34 patients similar to some patients with Bardet-Biedl syndrome (BBS) where ERG deficits precede retinal degeneration [38–40]. The diagnosis of BBS in patients is often delayed until an ERG is performed [39, 41]. Therefore, here, we will lie to call attention the need for use of detailed electroretinography to detect possibility of retinal disturbance in SCA34 patients in the future.

There is currently no cure for SCAs, and current therapies such as physical and speech therapy are directed at managing the symptoms [7]. Due to the heterogeneity of the causative mutations of SCA, targeted therapeutic approaches may be required for each genotype. Therefore, the deficiency in VLC-SFA and VLC-PUFA synthesis related to the L168S ELOVL4 variant suggests that repletion of these fatty acids may be a therapeutic approach specifically for SCA34 disease. As previously reported for other SCAs, targeting proteotoxicity, RNA toxicity, and ion channel dysfunction are also possible potential therapeutic targets [2, 25–29]. In SCA13, Purkinje cell hyperexcitability is thought to trigger cerebellar degeneration in patients with infant-onset SCA13 [42]. Similar to differences in the onset of SCA34 between L168S and L168F variants, infant- and adult-onset forms of SCA13 are caused by distinct mutations in the *Kv3.3* gene that encodes the Kv3.3 voltage-gated K^+^ channel [42]. Kv3.3, like ELOVL4, is expressed in cerebellar neurons such as Purkinje cells [42]. Interestingly, suppressing hyperexcitability of Purkinje neurons in SCA13 by using NS13001, an agonist of Ca2^+^-activated K^+^ channels known to reduce neuronal excitability, promoted early survival of neurons in zebrafish [42]. Similarly, oral treatment with NS13001 rescued Purkinje cell loss and gait function in SCA2 model mice [43]. It is, therefore, possible that SCA34-causing *ELOLV4* variants and impaired VLC-SFA synthesis may utilize similar mechanisms as in SCA13 to cause cerebellar atrophy.

## Conclusion

Here we report the first case of childhood-onset SCA34 with retinal dysfunction caused by a novel *ELOVL4* variant, L168S. The L168S variant caused a severe and rapid progression of cortical and cerebral atrophy with the patient losing her ability to walk at 4.5 years of age as well as retinal dysfunction and death within the first decade of life. Our biochemical analyses indicate that the L168S variant is deficient in making both VLC-SFA and VLC-PUFA products necessary for brain and retinal health [20, 25]. Therefore, depletion of VLC-FA may underlie the pathogenic mechanism of SCA34. Future studies to investigate the effect of the child-onset SCA34 caused by the L168S variant on cerebellar neurons compared to other adult-onset mutations, especially the L168F variant, would help us further understand how different *ELOVL4* variants affect VLC-FA biosynthesis to cause different tissue-specific disorders in humans. This understanding will enable the scientific community to explore potential therapeutic avenues to attenuating disease progression.

### Supplementary Information

**Additional file 1**: **Fig. 1A-F**. ERG performed at 4.5 years of age showed below normal and delayed responses to dark and light adapted ERGs that is indicative of a pan retinal defect in both the rod and cone systems with the rods having slightly greater dysfunction than cones.

**Additional file 2**: **Fig. 2A-F**. ERG performed at 6.5 years of age showed undetectable ERG responses for dark adapted ERG at 0.01 and 3.0 amplitudes. In addition, the light adapted 3.0 ERG was still reduced and delayed. Consistent with the 4.5 years ERG, the flicker amplitude was still normal but delayed.

## Data Availability

There are no associated datasets for these manuscripts. Related queries can be directed to the corresponding authors.

## Abbreviations

SCA34: Spinocerebellar ataxia-34
EKV: Erythrokeratodermia Variabilis
STGD3: Autosomal Stargardt-like macular dystrophy
VLC-PUFA: Very long chain polyunsaturated fatty acid
VLC-SFA: Very long chain saturated fatty acid
ELOVL: Elongation of very long chain fatty acid
ISCEV: International Society for Clinical Electrophysiology of Vision
ERG: Electroretinography
